# Association Between a Variable Number Tandem Repeat Polymorphism Within the DAT1 Gene and the Mesolimbic Pathway in Parkinson's Disease

**DOI:** 10.3389/fneur.2020.00982

**Published:** 2020-09-02

**Authors:** Stefan du Plessis, Minke Bekker, Chanelle Buckle, Matthijs Vink, Soraya Seedat, Soraya Bardien, Jonathan Carr, Shameemah Abrahams

**Affiliations:** ^1^Department of Psychiatry, Faculty of Medicine and Health Sciences, Stellenbosch University, Cape Town, South Africa; ^2^Stellenbosch University Genomics of Brain Disorders Research Unit, South African Medical Research Council, Cape Town, South Africa; ^3^Division of Molecular Biology and Human Genetics, Faculty of Medicine and Health Sciences, Stellenbosch University, Cape Town, South Africa; ^4^Departments of Developmental and Experimental Psychology, Utrecht University, Utrecht, Netherlands; ^5^Division of Neurology, Faculty of Medicine and Health Sciences, Stellenbosch University, Cape Town, South Africa

**Keywords:** DAT1, SLC6A3, Parkinson's disease, monetary incentive delay, orbitofrontal cortex, ventral striatum, functional magnetic resonance imaging

## Abstract

The loss of ventral striatal dopaminergic neurons in Parkinson's disease (PD) predicts an impact on the reward system. The ventrostriatal system is involved in motivational processing and its dysfunction may be related to non-motor symptoms such as depression and apathy. We previously documented that patients with PD had blunted Blood Oxygen Level Dependent functional magnetic resonance imaging (BOLD fMRI) reward task related activity during both reward anticipation (i.e., in the ventral striatum) and reward outcome (i.e., in the orbitofrontal cortex). Evidence for the modulation of brain function by dopaminergic genes in PD is limited. Genes implicated in dopamine transmission, such as the dopamine transporter gene (DAT1) may influence the clinical heterogeneity seen in PD, including reward processing. This study therefore sought to determine whether genetic differences in the DAT gene are associated with brain activity associated with response to reward in PD patients and controls. A sample of PD cases on treatment (*n* = 15) and non-PD controls (*n* = 30) from an ethnic group unique to South Africa were genotyped. We found a three-way interaction between GENOTYPE × BOLD fMRI REWARD × DIAGNOSIS [*F*_(1, 40)_ = 4.666, *p* = 0.037, partial η^2^ = 0.104]. PD patients with the DAT1 homozygous 10/10 repeat genotype showed a relative decrease in orbitofrontal cortex reward outcome related activity compared to the patient group who did not have this repeat. PD patients with other genotypes showed an expected increase in orbitofrontal cortex reward outcome related activity compared to controls. Given the small sample size of the PD group with the 10/10 repeat, these results should be considered preliminary. Nevertheless, these preliminary findings highlight the potential modulation of dopamine transporter polymorphisms on orbitofrontal reward system activity in PD and highlight the need for further studies.

## Introduction

Parkinson's disease (PD) is characterized by decreased dopaminergic availability in the brain, especially in the striatum (1). Changes in striatal dopaminergic tone in PD have been related to cognitive as well as reward processing abnormalities (2). Further, differences in fronto-striatal reward processing have been demonstrated in both medicated and unmedicated patients (2, 3). Specifically, blunted neural activity related to performance on a reward task was found to be a distinguishing factor in PD patients when compared to similarly aged controls (3). Brain activity patterns associated with reward may be linked to the non-motor symptoms of PD such as apathy (4) and impulsivity (5), and warrant investigation.

There is a growing body of literature documenting an association between candidate genes involved in the dopamine system and reward related brain activity (6–8). This includes the commonly occurring 10-repeat allele of the 40 bp variable number tandem repeat (VNTR) polymorphism within the Dopamine Active Transporter (DAT1) gene, also referred to as solute carrier family 6 member 3 (SLC6A3). Although the individual frequencies of the DAT1 repeats differ between ethnic groups, the 10-repeat allele is still the most common with frequencies ranging from 37 to 93% across several ethnic groups (9). The majority of *in vitro* studies have shown that the 10-repeat allele is associated with increased DAT1 expression in comparison to a commonly occurring 9-repeat (10–12). Increased DAT1 expression leads to increased activity of DAT1 and increased dopamine uptake, which results in a decrease of dopamine levels in the synapse, and this could potentially be related to poorer reward related activity in the ventral-striatal system. Using single photon emission computed tomography (SPECT), subjects homozygous for the DAT 10-repeat allele had a 22% relative increase in DAT protein availability in homozygous DAT 10-repeat homozygotes compared to those with the 9-repeat/10-repeat genotype (10). Using Quantitative real-time Reverse Transcription Polymerase Chain Reaction (RT-PCR), it was shown that increased levels of DAT1 expression were associated with the number of 10-repeat alleles (11). Radioligand binding and immunoblotting techniques also revealed statistically significant differences in DAT expression attributable to the DAT1 genotype, with lower the DAT1 density for the 9- and 10-repeat variants (12). Not all studies have found increased expression of DAT1 associated with this allele, with some reporting the opposite (13). Blood oxygen level dependent functional MRI (fMRI) nevertheless demonstrated relatively decreased reward anticipatory activity in the ventral striatum as well as decreased reward outcome related activity in the orbitofrontal cortex during a Monetary Incentive Delay (MID) reward processing task in individuals with the DAT1 10/10 repeat, compared to those with the 9/9 repeat (6). The MID is known to robustly activate the ventral striatum during reward anticipation and orbitofrontal cortex during positive reward outcome (14). This finding was replicated in another reward processing study examining orbitofrontal and ventral striatal activity in DAT1 10/10-repeat vs. 9/9 repeat carriers, showing greater responses to smoking vs. non-smoking cues (15). However, there were contrasting findings in a study examining the impact of genotype on reward processing thought to underlie long-term memory formation where the DAT1 10-repeat homozygotes demonstrated increased striatal activity compared with 9/10-repeat heterozygotes (8).

As the relationship between DAT1 genotypes and reward processing is further clarified, it is important to examine the impact that the DAT1 genotype could have on the course of illnesses such as PD, where disease related alterations in dopamine tone are particularly evident (16). To our knowledge, there are no studies that have examined the relationship between the DAT1 genotype and abnormalities in brain activation associated with reward processing in PD.

Previously, we identified a relative decrease in both anticipatory activity seen in the ventral striatum and reward outcome related activity in the orbitofrontal cortex in PD (3). Here, we aim to investigate the potential modulating effects of the DAT1 10/10 genotype on these brain changes in a subsample of 15 PD patients and 30 matched healthy controls, drawn from a larger cohort, who were genotyped and underwent fMRI whilst performing a monetary incentive delay task (17, 18). Given the aforementioned findings in the literature, we predicted that PD patients relative to controls, with the DAT1 10/10 repeat genotype, compared with DAT1 heterozygotes, would have the lowest levels of reward related activity in both the ventral striatum during anticipation and in the orbitofrontal cortex during reward outcome.

## Materials and Methods

The study participants form part of a larger cohort examining the genomic and environmental signatures that are common to PD, Posttraumatic Stress Disorder, Schizophrenia and metabolic syndrome (named the “Shared Roots” study, MRC-RFA-UFSP-01-2013). The study has been approved by Health Research Ethics Committee (HREC N13/08/115) of Stellenbosch University, Tygerberg Hospital, Cape Town, South Africa, with annual renewal.

All participants were recruited from the same geographical region in Cape Town, South Africa, were unrelated and matched to socioeconomic status (lower to middle income status). All self-identified as “mixed ancestry” which refers to an ethnic population unique to South Africa and resulting from an admixture of individuals of African, European and Asian ancestral origins (19). This is the first published report on *DAT1* genotypes in a South African Mixed Ancestry population. A diagnosis of PD was clinically confirmed by a neurologist according to MDS diagnostic criteria (20). A healthy (non-PD) control group was recruited and matched for ethnicity. Controls did not have current significant psychopathology or other significant confounding medical conditions.

### Clinical Assessments

All participants received a full clinical examination. They were screened for any confounding psychopathology using the Mini-International Neuropsychiatric Interview (MINI version 6.0.0). The Unified Parkinson's Rating Scale (UPDRS) (Version 3.0) was completed for the PD patients (21). Handedness was determined by the Edinburgh Handedness Inventory (22). All participants were asked to take their PD medication as normal, prior to scanning. All participants received a urine drug screen immediately before their MRI scan. Participants with severe head injury, confounding intra-cranial pathology, current severe psychopathology and/or drug abuse and other medical conditions that could confound behavioral as well as fMRI measures were excluded.

### Genotyping of 40 bp DAT1 VNTR Polymorphism

Venous whole blood was collected from all study participants for the genetic analyses. Genomic DNA was isolated with the use of an in-house phenol/chloroform method prior to 2016 and a salting-out precipitation method (Gentra Puregene Blood Kit), for samples collected post 2016. Polymerase chain reaction (PCR) amplification was performed using primers DAT1 forward: 5′-ATGGGGGTCCTGGTATGTCT-3′ and reverse: 5′- GGCACGCACCTGAGAGAAAT-3′; that were designed using OligoAnalyzer (www.idtdna.com/oligoanalyzer), BLAST (www.ncbi.nlm.nih.gov/BLAST/) and primerBLAST (www.ncbi.nlm.nih.gov/tools/primer-blast/) for optimal binding to the region of interest. PCR was performed in 25 μl reactions which contained 0.4 μM DAT1 forward and reverse primers (Inqaba biotec™, South Africa), 0.075 μM of each dNTP, 0.25 U GoTaq® G2 Flexi DNA Polymerase, 1x Colorless GoTaq® Flexi Buffer, 1.5 mM MgCl_2_ solution (Promega, Madison, WI, USA) and 30 ng genomic DNA. The PCR conditions comprised of: initial denaturation at 95°C for 10 min; followed by 35 cycles of denaturation at 93°C for 1 min, annealing at 58°C for 30 s and extension at 72°C for 1 min; and a final extension step at 72°C for 10 min using a thermocycler (Applied Biosystems, GeneAmp® PCR System 2700, Singapore). The PCR product was visualized using electrophoresis on a 1% SeaKem® LE Agarose gel (Consort Electrophoresis Power Supply, 800 Series, E844, Belgium). Genotyping was carried out by comparing the size of the PCR product, visualized on the agarose gel, to the expected product size determined based on the reference DNA sequence (NM 001044.5) in Ensembl (www.ensembl.org/index.html).

### Monetary Incentive Delay (MID) fMRI Paradigm

**Figure 1:**
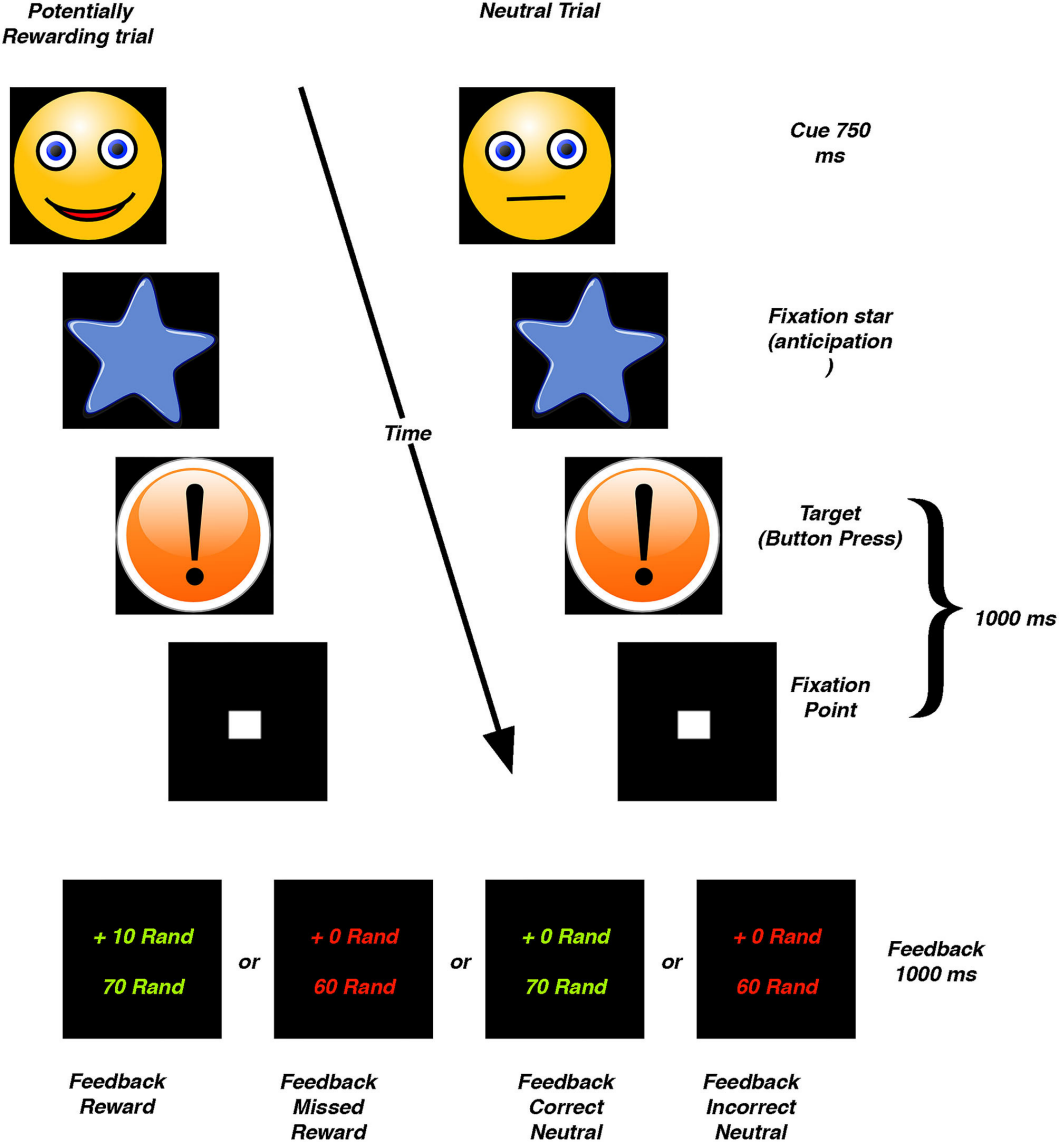
Schematic representation of the Monetary Incentive Delay task. Rand, South African Rand (ZAR).

All participants performed a modified version of the MID task (23). To enhance task comprehension, as well as keep the number of scan acquisitions to a minimum, only reward and neutral cues were used in this task. The task is described in detail elsewhere (23). Briefly, during each scan trial participants were required to respond as rapidly as possible when a target cue was presented. A smiling face immediately preceded the target, to indicate a potentially rewarding trial, and a neutral face was presented prior to neutral trials. After seeing the face cue, a blue star was shown for a short pseudo random interval immediately followed by the target cue (i.e., reward anticipation). If a participant responded in time to the target cue, a screen with green lettering appeared indicating the total reward won (i.e., reward outcome). If a participant did not respond in time, red letters appeared. During reward trials, the monetary reward was incrementally increased (fixed increments of ZAR10) (see Figure 1).

The reward anticipation period as well as the inter-trial interval were “jittered” to reduce collinearity between reward anticipation and reward outcome (mean duration 3,286 ms, range 779–6,729 ms; mean duration 3,535 ms, range 1,029–6,979 ms, respectively). The reward outcome period was 2,000 ms per trial. The entire task therefore consisted of 60 trials, with a mean duration of 9,571 ms (range 4,946–16,107 ms), resulting in a total task duration of 9 min 35 s.

To ensure an equal number of rewarded and unrewarded trials, the duration of the target cue was adapted to the fastest response time of the participant during a training session. By matching task performance across subjects in this way, we controlled for differing levels of performance across the groups. The target score was set to approximately ZAR150 (~10 USD) for each group.

### Behavioral Data Analysis

Neutral correct trials and rewarded trials were compared between case-control (diagnostic) and DAT1 genotype groups using a repeated measures analysis of variance (RMANOVA), modeling for REWARD (i.e., neutral correct vs. rewarded trials) × DIAGNOSIS (i.e., PD and controls) × GENOTYPE (i.e., DAT1 10/10 repeat vs. other genotypes) interaction effects. Monetary reward across diagnostic and genotype groups was compared with a standard *t*-test. If the 3-way modeling for REWARD × DIAGNOSIS × GENOTYPE interaction was significant, *post-hoc* testing was performed to identify whether the 3-way interaction was driven by disease status or genotype.

### Image Acquisition

Scans were acquired on a 3T Siemens Allegra at the Combined Universities Brain Imaging Center (CUBIC). A total of 360 whole-brain 2D-EPI images (TR = 1,600 ms, TE = 23 ms, flip-angle: 72.5 degrees, FOV: 256 ×256, 30 slices, 4 mm isotropic voxels) were acquired in 9 min 35 s. For image registration, a T1 ME-MPRAGE weighted structural scan was acquired (TR = 2,530 ms; TE1 = 1.53 ms TE2 = 3.21, ms, TE3 = 4.89 ms, TE4 = 6.57 ms, flip-angle: 7 degrees, FoV: 256 mm, 128 slices, 1 isotropic voxel size) (24).

### Image Pre-processing

Images were analyzed using SPM12 (http://www.fil.ion.ucl.ac.uk/spm/software/spm12/). Pre-processing and first-level statistical analysis was undertaken as previously described (3). In brief, pre-processing involved correction for slice timing differences, re-alignment to correct for head motion, spatial normalization to the Montreal Neurological Institute template brain, and spatial smoothing to accommodate inter-individual differences in neuro-anatomy. Head motion parameters were analyzed to ensure that the maximum motion did not exceed a predefined threshold (scan-to-scan >2 mm).

### First Level fMRI Statistical Analysis

The pre-processed time-series data for each participant was analyzed using a standard general linear model (GLM) analysis. The model consisted of six factors of interest, representing haemodynamic changes time-locked to trial periods of (1) anticipation of receiving a potential reward, i.e., during and after the presentation of the reward cue (reward anticipation), (2) the lack of reward anticipation during and after a neutral cue (neutral anticipation), (3) feedback reflecting when money was received for a successful reward trial (reward outcome), (4) feedback when no reward was received, (5) feedback reflecting when the button was pressed in time during a neutral trial (neutral correct outcome), and (6) feedback reflecting an incorrect response in a neutral trial, i.e., when the target was missed when no reward was offered (Figure 1). The onset of the factors modeling anticipation (duration range 1,529–7,479 ms) was at the presentation of the cue, while the onset of the factors modeling feedback (duration: 2,000 ms) was at the presentation of the target, including the button press to the target and subsequent feedback (see Figure 1). Motion parameters from the realignment procedure were included as factors of no interest. Low frequency drifts were removed from the signal by applying a high-pass filter with a cut-off frequency of 128 Hz.

### Region of Interest Analyses

Primary analyses were performed in one region of interest (ROI): the combined bilateral ventral striatum for anticipation, and combined bilateral orbitofrontal cortex for reward outcome, based on previous findings (23). These regions were defined using the Automated Anatomical Labeling (AAL)-atlas (25) and the Oxford-GSK-Imanova Striatal Connectivity Atlas for the ventral striatum (26). For each participant, the mean activation level (expressed as percent signal change) during the contrasts of interest specific to reward anticipation and reward outcome (reward anticipation, neutral anticipation, reward outcome, and neutral correct outcome) was averaged over all the voxels of each ROI using SPM12 and custom MATLAB R2019a scripts.

Similar to the behavioral data analysis, these values were used in a RMANOVA, testing for main and group effects in activation levels between neutral vs. potentially rewarding trials, reward anticipation vs. reward outcome, and correct neutral trials vs. positive reward outcome. As in the behavioral analysis, we modeled for a REWARD × DIAGNOSIS × GENOTYPE interaction effect.

## Results

The Shared Roots cohort comprised 81 PD patients and 79 controls. All participants were genotyped for the 40 bp DAT1 VNTR. Data was originally collected from 2 separate scan sites. Due to the low number of controls relative to patients available at the second site (*n* = 6 with DAT1 10/10 genotype, *n* = 2 with other genotypes) which resulted in an unbalanced sample for the second scan site, we chose only to include data from the first scan site. Of these, 18 patients and 39 controls had fMRI, T1 structural scan and genotype data. Three PD patients and seven controls were excluded due to the presence of motion or other scanner related artifacts. Two controls were excluded due to poor task performance. This resulted in a final sample of 15 patients and 30 controls. The demographics of the 45 study participants are reflected in Table 1. There was a small but significant difference in age between the cases and controls. We therefore included age as a covariate in all analyses. Although there were significantly fewer females present in the patient group than in the control group, we chose not to correct for this in our final model, as we found no sex-based differences on the MID in our larger sample (3, 18). Patient and control groups were also matched in terms of several important motion parameters (Table 1) (27).

**Table 1:** Demographics of the 45 study participants.

	**Patients**		**Controls**			
	***n*** **=** **15**		***n*** **=** **30**			
	**Mean**	**SD**	**Mean**	**SD**	**Test score**	***p*-value**
Age	61.59	9.56	56.56	6.59	*t* = 2.070	0.04^*^
Sex (M/F)	11/4		12/18		X^2^ = 4.447	0.04^*^
Handedness (R/L)	14/1		29/1		X^2^ = 0.262	0.61
Months since diagnosis	56.6	41.92				
LED (mg/day)	560	307.76				
Hoehn & Yahr staging	2.57					
ADL (best/worst)	73.33/65.33					
Reward Won (ZAR)	118.67		121.67		*t* = −0.309	0.76
**fMRI motion parameters**						
Mean motion	0.09	0.03	0.10	0.03	*t* = −1.141	0.26
Maximum motion	0.47	0.28	0.52	0.31	*t* = −0.517	0.61
Total number of movements	124.87	80.20	138.27	64.75	*t* = −0.604	0.55
Mean rotation	0.001	0.0003	0.001	0.0004	*t* = −0.867	0.39
Maximum rotation	0.006	0.0025	0.008	0.0070	*t* = −0.803	0.43

* *Significant at p < 0.05 level*.

**Figure 2:**
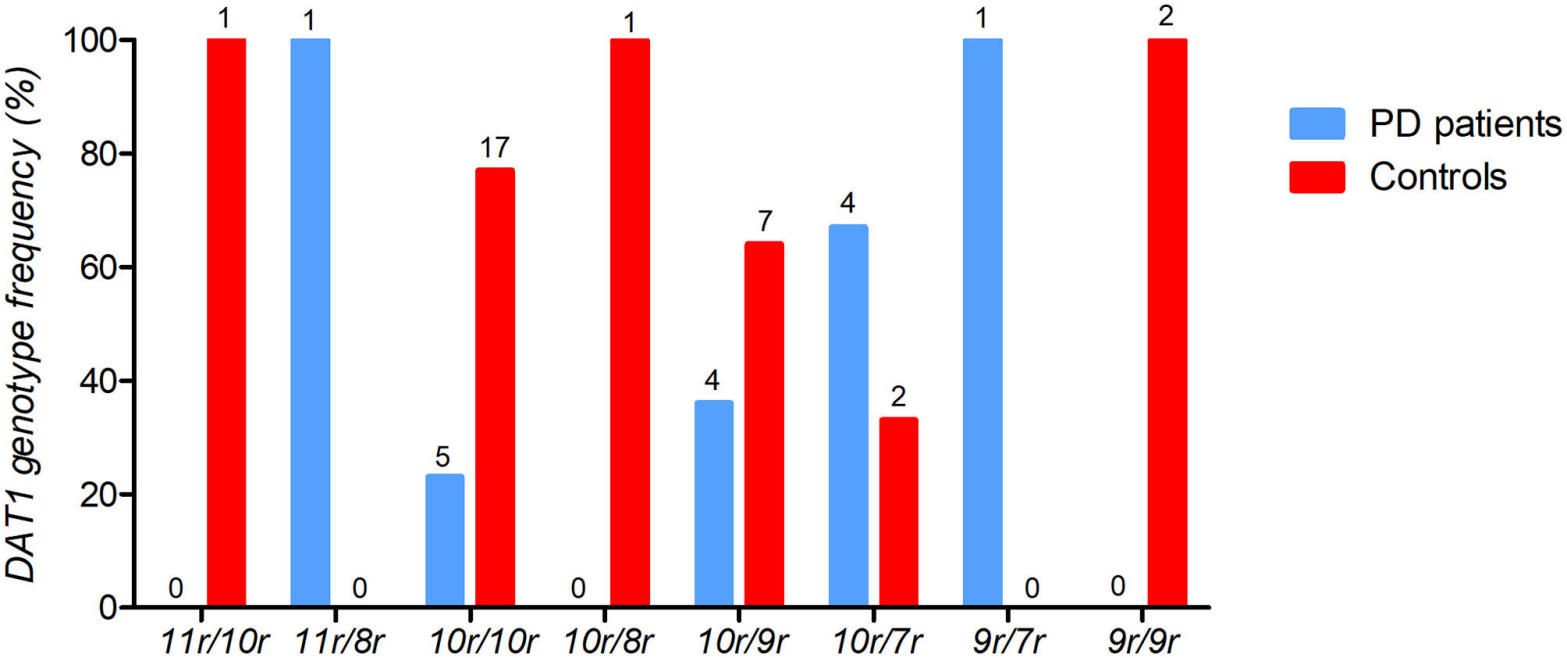
The frequencies of *DAT1* variable number tandem repeat (VNTR) for PD patients (*n* = 15) and healthy controls (*n* = 30). The percentage of *DAT1* VNTR frequencies are depicted with the individual counts (*n*) displayed above each genotype bar.

Similar to European cohorts, the DAT1 10/10 repeat was the most commonly occurring repeat in the sample (45.8%), followed by the 10/9 repeat. Interestingly, our sample contained few DAT1 9/9 repeats (4.2%, see Figure 2) unlike cohorts of European ancestry where it is often frequent. Importantly, there was no significant difference in the frequencies of the genotype subgroups, which were balanced between patients and controls (X^2^ = 2.179, *p* = 0.14). As we had an *a priori* hypothesis for the DAT1 10/10 repeat and had observed a relatively low frequency of the other repeats, we divided the sample into two groups: a “10/10 repeat” genotype group compared to all the other genotypes.

### No Difference Observed in Behavioral Data

Since the task was adjusted according to participant's performance level, patients and controls received an equal amount of reward [*t*_(43)_ = −0.309, *p* = 0.759]. Both patients and controls appropriately responded more rapidly to rewarded trials [*F*_(1, 41)_ = 5.633, *p* = 0.022], but there was no REWARD × GENOTYPE interaction effect [*F*_(1, 41)_ = 0.103, *p* = 0.749].

### No Reward Anticipation Effect Observed in the Ventral Striatum

Contrary to our hypothesis, we found no main effect for reward anticipation in the ventral striatum [*F*_(1, 41)_ = 1.615, *p* = 0.211], nor a DIAGNOSIS interaction effect [*F*_(1, 41)_ = 2.227, *p* = 0.143].

### Reward Outcome Effect Observed in the Orbitofrontal Cortex

**Figure 3:**
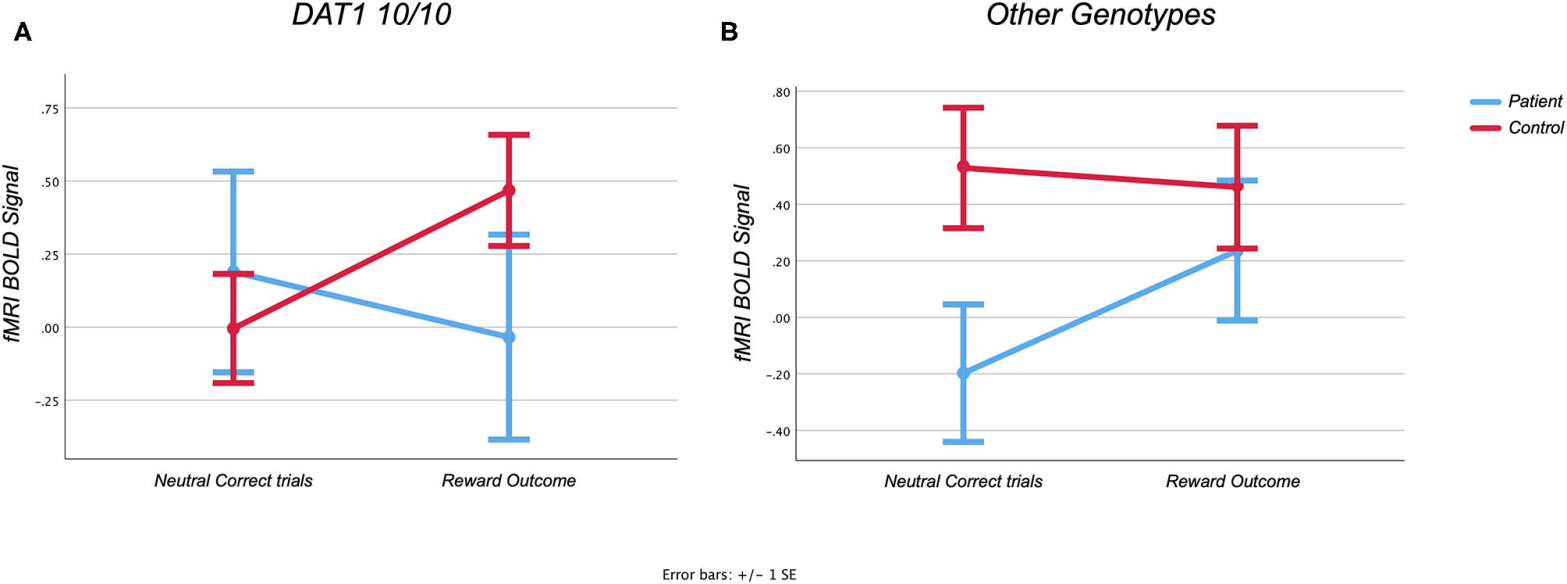
**(A,B)** Region of interest analysis of the Monetary Incentive Delay task. Graphs showing task related activity during the Monetary Incentive Delay task in the orbitofrontal cortex during reward feedback.

Although there was no main effect for reward outcome in the orbitofrontal cortex [*F*_(1)_ = 1.172, *p* = 0.285], there was a three-way REWARD × DIAGNOSIS × GENOTYPE interaction effect [*F*_(1, 40)_ = 4.666, *p* = 0.037], while controlling for age, in line with our hypothesis (see Figure 3). As predicted, after *post-hoc* testing, Parkinson's patients who have the DAT1 10/10 genotype demonstrated a decrease on average in bold signal from neutral to rewarded trials, whereas those with other genotypes demonstrated a normal increase in activity [*F*_(1)_ = 1.678, *p* = 0.22, partial η^2^ = 0.123). Interestingly, controls demonstrate the opposite effect, showing a relative increase in those that have the DAT1 10/10 repeat, with an absent response for the other genotypes [*F*_(1)_ = 3.017, *p* = 0.094, partial η^2^ = 0.101]. Uncorrected *post-hoc* testing however failed to yield significant results. A marginally larger effect was noted when comparing patients with and without the DAT1 10/10 genotype (partial η^2^ = 0.123), than when comparing controls with and without the genotype (partial η^2^ = 0.101). This suggests that the three-way interaction effect in our main analysis was driven more by the patients with and without the DAT1 10/10 genotype relationship. As *post-hoc* testing could not adequately distinguish between the various subgroups and given the small sample size, particularly that of the Parkinson's group with the DAT1 10/10 repeat (*n* = 5), these results should be considered exploratory.

The blunted response in the control group which did not have the DAT1 10/10 repeat can still be expected for this age group. Results remained unchanged, however, when the age covariate was removed. A sample using a wider age range would be needed to adequately address this question.

### Exploratory Whole Brain Voxel Wise Analysis

As we had a specific hypothesis, directed at specific brain regions we did not focus on whole brain analyses. Whole brain exploratory analysis did not yield any additional information.

## Discussion

We investigated the potential genetic underpinnings of reward processing abnormalities seen in PD compared to healthy controls. As predicted, we found evidence for poorer reward outcome-based reactivity in the orbitofrontal cortex for patients with the DAT1 10/10 genotype compared to those with other genotypes. Contrary to our hypothesis, we found no such differences in the ventral striatum during reward anticipation. To our knowledge this is the first-time reward-related functional genotypes have been investigated in PD.

Our finding that DAT1 10/10 carriers had abnormal reward outcome related activity in the orbitofrontal cortex could reflect an increased vulnerability for PD-related dopaminergic cell loss in this region. Indeed, it has been shown that the DAT1 10/10 genotype is associated with lower synaptic dopamine availability due to possible increased levels of dopamine transporters (10). Not all studies reported this however, with some suggesting the opposite (13). Our results corroborate a potential hypo-dopaminergic state in the DAT1 10/10 group, as BOLD fMRI activity has been found to correlate with dopamine reactivity in this region (14). However, further exploration of this link with more direct methods such as the use of positron emission tomography (PET) would be needed to confirm this. Although the DAT1 10/10 genotype is one of the more common functional variants reported in the literature, it is possible that there are other unexamined variants that are stronger predictors of dopaminergic hypofunction. Our results confirm that DAT1 10/10 is a potential predictor of reward function variability in diseased states. As our present sample only includes patients on treatment, we cannot account for potential treatment effects. It could be that PD medication has an impact on the normal dopaminergic tone of the ventral tegmental area, which could also have a differential impact across genotypes. As our patients were not assessed while medication free and dopamine activity was not directly measured, we cannot comment on treatment effects. Further treatment effect studies are advised. Interestingly, although Parkinson's patients without the 10/10 DAT1 repeat showed a normal increase in reward outcome related activity, controls without the 10/10 repeat showed a relatively flattened out response. The absence of a response in the controls could be age related, as a similar flattened out response has been observed in similarly aged healthy controls in previous studies (3, 18). The relative increase in reward outcome related activity Parkinson's patients without the DAT 1 10/10 repeat could possibly reflect treatment effects. Again, future studies investigating medication and genotype interaction effects in larger number of patients and controls are needed to substantiate this finding.

Contrary to our previous findings, we did not find any reward anticipatory related activity in the ventral striatum in this particular sub-sample, nor any effect of genotype in this region for this subgroup. The absence of a reward anticipation effect could be explained by poor data quality or poor task comprehension. This was unlikely to be the case in our study as our groups also showed low levels of motion and did not differ on important measures of motion. All groups also demonstrated good task comprehension, as they increased their response times appropriately during rewarded trials. Another potential explanation is the relative older age of the current subsample, which could explain the general lack of signal for this region. Indeed, it has been found that ventral striatal but not orbito-frontal activity *per se*, decreased with normal aging (18).

Although our findings do indeed substantiate our hypothesis that reward related functioning is related, at least in part, to DAT1 genotype, our sample is small, and therefore these findings should be considered as exploratory. Although we found significant differences in the orbitofrontal regions, we cannot completely rule out similar findings for the ventral striatum due to our limited sample size. Furthermore, although our study supports functional associations with the commonly occurring *DAT1* genotype, this does not necessarily mean that it is the only or even the best predictor of reward related functioning. Future, larger studies should also explore other dopamine-related genes such as catechol-O-methyltransferase (*COMT*) (6).

Despite these limitations, our study has important implications. Reward related function loss, and PD non-motor symptoms by extension, could be exacerbated in certain vulnerable genotypes. This should be considered in future studies of genetic vulnerability and treatment in PD. Genetic risk factors could potentially play an important role in the non-motor symptoms of PD.

## Data Availability Statement

The raw data supporting the conclusions of this article will be made available by the authors, without undue reservation, to any qualified researcher.

## Ethics Statement

The studies involving human participants were reviewed and approved by Health Research Ethics Committee (HREC N13/08/115) of Stellenbosch University, Tygerberg Hospital, Cape Town, South Africa. The patients/participants provided their written informed consent to participate in this study.

## Author Contributions

SP: study design, fMRI task administration supervision, data processing, analysis, interpretation, and lead author on manuscript preparation. MB: genetic samples analysis, interpretation, and manuscript preparation. CB and MV: fMRI data processing, analysis, and interpretation. SS, SB, and JC: study design, interpretation, and manuscript preparation. SA: genetic sample analysis and interpretation. All authors contributed to the article and approved the submitted version.

## Conflict of Interest

The authors declare that the research was conducted in the absence of any commercial or financial relationships that could be construed as a potential conflict of interest. The handling editor declared a past collaboration with the authors.
